# Feasibility assessment of the Eye Scan Ultrasound System for cataract characterization and optimal phacoemulsification energy estimation: protocol for a pilot, nonblinded and monocentre study

**DOI:** 10.1186/s40814-022-01173-2

**Published:** 2022-09-29

**Authors:** Lorena Petrella, Sandrina Nunes, Fernando Perdigão, Marco Gomes, Mário Santos, Carlos Pinto, Miguel Morgado, António Travassos, Jaime Santos, Miguel Caixinha

**Affiliations:** 1 Department of Electrical and Computer Engineering, Univ Coimbra, 3030-290 Coimbra, Portugal; 2 Centre for Mechanical Engineering, Materials and Processes, Univ Coimbra, 3030-788 Coimbra, Portugal; 3 Centre for Informatics and Systems of the University of Coimbra, Univ Coimbra, 3030-290 Coimbra, Portugal; 4 Coimbra Institute for Clinical and Biomedical Research, Univ Coimbra, 3000-548 Coimbra, Portugal; 5 Instituto de Telecomunicações, 3030-290 Coimbra, Portugal; 6 Coimbra Institute for Biomedical Imaging and Translational Research, Univ Coimbra, 3004-516 Coimbra, Portugal; 7 Department of Physics, Univ Coimbra, 3004-516 Coimbra, Portugal; 8 Coimbra Surgical Centre, 3045-089 Coimbra, Portugal; 9 Department of Physics, Univ Beira Interior, 6291-001 Covilhã, Portugal

**Keywords:** Cataract, Ultrasound, Diagnosis, Phacoemulsification surgery

## Abstract

**Background:**

Cataracts are lens opacifications that are responsible for more than half of blindness cases worldwide, and the only treatment is surgical intervention. Phacoemulsification surgery, the most frequently performed cataract surgery in developed countries, has associated risks, some of which are related to excessive phacoemulsification energy levels and times. The protocol proposed in herein will be used to evaluate the feasibility of a new experimental medical device, the Eye Scan Ultrasound System (ESUS), for the automatic classification of cataract type and severity and quantitative estimation of the optimal phacoemulsification energy.

**Methods:**

The pilot study protocol will be used to evaluate the feasibility and safety of the ESUS in clinical practice. The study will be conducted in subjects with age-related cataracts and on healthy subjects as controls. The procedures include data acquisition with the experimental ESUS, classification based on the Lens Opacity Classification System III (LOCS III, comparator) using a slit lamp, contrast sensitivity test, optical coherence tomography, specular microscopy and surgical parameters.

ESUS works in A-scan pulse-echo mode, with a central frequency of 20 MHz. From the collected signals, acoustic parameters will be extracted and used for automatic cataract characterization and optimal phacoemulsification energy estimation.

The study includes two phases. The data collected in the first phase (40 patients, 2 eyes per patient) will be used to train the ESUS algorithms, while the data collected in the second phase (10 patients, 2 eyes per patient) will be used to assess the classification performance. System safety will be monitored during the study.

**Discussion:**

The present pilot study protocol will evaluate the feasibility and safety of the ESUS for use in clinical practice, and the results will support a larger clinical study for the efficacy assessment of the ESUS as a diagnostic tool. Ultimately, the ESUS is expected to represent a valuable tool for surgical planning by reducing complications associated with excessive levels of phacoemulsification energy and surgical times, which will have a positive impact on healthcare systems and society. The study is not yet recruiting.

**Trial registration:**

ClinicalTrials.gov identifier NCT04461912, registered on July 8, 2020.

## Introduction

### Background

Cataracts account for over half of blindness cases worldwide, with the only established treatment being the surgical removal of the cataractous lens and replacement with an intraocular lens (IOL) [1, 2]. Several scientific contributions were crucial to the evolution of cataract surgery. The first key finding was identified in the mid-twentieth century for Second World War fighter pilots, in which intraocular fragments of shattered acrylic from canopies were well tolerated. This fact led to the development of the first IOL using this material [3]. In approximately 1960, A-scan ultrasonic systems emerged for measuring the axial eye length [4], which allowed to determine the refractive power of the IOLs, thereby reducing the previously significant postoperative refractive errors [3, 4]. In 1967, high-precision ultrasonic equipment working at 10 MHz was further developed, which solved some remaining limitations of the previous systems, such as the deformation produced by the probe over the cornea, inability to perform alignments within the visual axis and large beam diameters [5]. Moreover, in the same year, the first phacoemulsification surgery was performed using ultrasonic waves for fragmentation of the cataractous lens [6]. This technique introduced a more controlled, faster and safer method of extracapsular cataract extraction, which provided significant advantages for cataract treatment.

Cataract surgery has evolved in recent years, thus making the method safer and more efficient. However, risks are still associated with this procedure. Among them, posterior lens capsular rupture and corneal endothelial cell loss related to excessive levels of phacoemulsification energy represent approximately 5 to 10% of postoperative complications [7, 8]. Posterior capsular rupture has diverse associated risks such as vitreous loss and dropped nucleus, increasing the risk of cystoid macular oedema or retinal detachment. In turn, corneal oedema and opacification may result from corneal endothelial cell loss. These complications may limit or prevent visual recovery [7].

Acoustic parameters extracted from ultrasonic waves propagating in cataractous lenses have been widely studied to characterize their structural and biomechanical properties. Several studies have characterized the presence and severity of cataracts using this approach [4, 8–12]. Moreover, studies have used ultrasound for cataract hardness estimation [8, 13–20] since the efficient energy that must be applied to phacoemulsification surgeries is directly related to cataract hardness [17]. Most of these studies were conducted in porcine lenses due to the similarities in their acoustic parameters with human lenses [21].

Preclinical studies were conducted by the research team with two models of cataracts: ex vivo in porcine lenses [8, 20] and in vivo in rat lenses [12]. These studies evaluated several automatic classification methods for cataract hardness and severity based on features extracted from the ultrasonic signals. The results showed a precision, sensitivity and specificity of 99.7% for cataract severity classification, as well as a statistically significant difference in lens hardness for the different cataract severities [12].

The present pilot study protocol aims to evaluate the Eye Scan Ultrasound System (ESUS), which was developed in the preclinical studies [22] and adapted for clinical applications [23] in human lenses in vivo. Because it is a first in human study, and to reduce risks and optimize human and financial resources, a pilot protocol was designed for feasibility assessment of the ESUS in clinical practice. It aims to assess the feasibility of detecting and characterizing human cataracts by ultrasound and the suitability and safety of the technique for use in clinical practice. Related to cataract characterization, the study protocol was designed to evaluate the performance of the experimental medical device ESUS for the automatic classification of cataract type and severity based on the Lens Opacity Classification System III (LOCS III, [24]) and to estimate the optimal phacoemulsification energy (OPE).

The study protocol is organized in two phases. In the first phase, the collected data will be used for training the automatic detection and classification algorithm for human cataracts. In the second phase, the collected data will be used for a preliminary assessment of the algorithm performance. Cataracts classified according to the LOCS III for type and severity [24] will be used as comparators. Safety will be evaluated throughout the study.

The experimental medical device may represent an important advance in cataract treatment based on two main contributions. First, cataracts should be detected at incipient stages, even before the onset of symptoms, in patients at high risk of developing cataracts. At incipient stages, visual acuity is preserved, which may favour the outcomes of emerging pharmacological therapies [1, 25]. On the other hand, with ESUS, it is expected to achieve quantitative and automatic estimation of OPE. It may represent a valuable tool in surgical planning by reducing complications associated with excessive levels of phacoemulsification energy and excessive surgical times [7]. These advances are relevant not only for patients but also for public health systems because they may reduce waiting lists and associated costs.

Further clinical studies will be needed to enlarge the database to improve the performance of cataract classification and better evaluate the technique’s efficacy and to include other causes of cataracts since only age-related cataracts are considered in the present protocol.

The manuscript follows the SPIRIT [26] and CONSORT [27] reporting guidelines and checklists and the editorials [28, 29].

### Objectives

#### Primary objective

Evaluate the feasibility of the ESUS (experimental medical device) for cataract characterization in humans.

#### Secondary objectives

i. Automatically detect and classify cataracts according to their type and severity.
ii. Detect cataract in the incipient stage.
iii. Estimate the OPE.
iv. Assess the automatic classification performance.
v. Evaluate the safety of the ESUS.

## Methods

### Trial design

Pilot, nonblinded and monocentre study.

### Participants

#### Study setting

The participants will be recruited from patients who have a medical consultation scheduled at the clinical centre (Coimbra Surgical Centre, Portugal).

Two groups are considered in this study. For the cataract group, the patients will be invited to participate after being diagnosed with cataracts and referred to phacoemulsification surgery by the physician. For the control group, the patients will be invited to participate after the presence of cataracts is excluded. For both groups, the inclusion/exclusion criteria must be verified.

#### Inclusion and exclusion criteria

##### Inclusion criteria

i. Between 50 and 70 years old
ii. Either gender
iii. Cataract group

- ○ Patients with age-related cataract
- ○ Indication for phacoemulsification surgery

iv. Control group

- ○ Patients without cataract

xxii. Signed consent

##### Exclusion criteria

i. The presence of any other ophthalmological condition or systemic disease that could affect the results.

#### Recruitment procedure and informed consent

Medical investigators of the research team will recruit the participants. He/she must inform the patients about the study objectives, the procedures involved, the visit schedule, the benefits and risks of their participation, the privacy policies and the freedom to participate or withdraw. The investigator must provide enough time for consent form reading and clarify all doubts. If the patient agrees to participate, he/she (or their legal representative) and the medical investigator must sign the consent form. One copy of the consent form will be given to the participant, and the original document will be kept at the clinical centre.

#### Participation time and anticipated withdrawal

The maximum participation time for each subject is 3 months. Participant inclusion will be confirmed on the same day that cataracts are diagnosed or excluded. Participants in the control group will complete the procedures on the same day, and a remote follow-up will be conducted 1 week later. Participants in the cataract group will complete the study on the day of the second postoperative follow-up (1 month after surgery). If the second eye receives an indication for phacoemulsification surgery, data for this second eye will also be collected.

An anticipated withdrawal of the participants is considered in the following situations: the occurrence of any unanticipated adverse effects, anticipation of a risk situation, any physical condition that makes it impossible to conduct the procedures involved in the study, or the voluntary withdrawal of the participant. In all these cases, the causes for participant withdrawal should be registered in the individual case report form (CRF), and the participants will be encouraged to perform a final follow-up. Codified data collected before the participant withdrew can be used for the purpose of the study.

#### Sample size and allocation

In the first phase of the clinical study, forty participants will be included, and the two eyes will be studied. The participants will be equally distributed into four groups (based on slit lamp/LOCS III classification): participants with incipient, moderate and severe cataracts and participants without cataracts will be included as the control group. The second phase will include at least ten participants, and the two eyes will be studied. Safety will be evaluated for all the recruited participants along the first and second phases.

Since this is a protocol for a pilot study, no sample size estimate was performed. The number of eyes expected to be analysed along the study (i.e. 20 per arm) was considered adequate for this study [30]. The statistical power will be determined at the end of the study for statistically significant results.

### Procedures

The participants’ visit schedule is presented in Table 1, and descriptions of the interventions and assessments are given below.

**Table 1:** SPIRIT figure showing the schedule of enrolment, interventions and assessments

	Enrolment	Allocation	Post-allocation	Post-allocation			
Time point (day)	t_1_			t_2_	t_3_	t_4_	t_5_
Enrolment							
Demographic and medical record	✓						
Slit lamp and LOCS III	✓						
Inclusion/exclusion criteria	✓						
Informed consent	✓						
Allocation		✓					
Interventions							
Phacoemulsification surgery^b^					✓		
Assessments							
ESUS			✓				
Contrast sensitivity^a^			✓		✓		
Specular microscopy^b^			✓			✓	
Optical coherence tomography^b^			✓			✓	✓
Surgical and postoperative complications record^b,c^					✓	✓	✓
Safety monitoring^d^			✓	✓			
Adverse events			✓	✓	✓	✓	✓

t_1_, diagnosis, recruitment and ESUS procedure; t_2_, safety assessment (a week after t_1_); t_3_, phacoemulsification surgery; t_4_, 1st follow-up (a week after t_3_); and t_5_, 2nd follow-up (a month after t_3_)

^a^Procedure to be performed alternatively on t_1_ or t_3_

^b^Procedures to be conducted only on cataract group

^c^If both eyes received indication for phacoemulsification surgery, data from both surgeries will be collected

^d^A clinical evaluation will be done immediately after the ESUS study and a remote evaluation 1 week later. The procedures will be performed in both eyes

#### ESUS (investigational medical device)

**Fig. 1:**
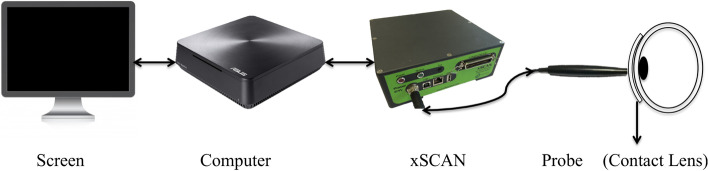
Diagram of the main ESUS components

The ESUS components are presented in Fig. 1 and Table 2. The system is composed of an A-scan ophthalmic probe working in pulse-echo mode, with an acoustic working frequency of 20 MHz and focal distance of approximately 8 mm. The active probe surface is shaped to match the cornea curvature, thus favouring their coupling and signal transmission with minimal coupling pressure. The probe front face (that contacts the eye) is manufactured with biocompatible material. The probe is connected to the xScan, whose functions are as follows: send excitation pulses to the probe, generate synchronism signals, digitize the signals captured back by the probe (at a sample frequency of 100 MHz) and preprocess the digitized signals (filtering and amplification). The output of the xScan system is connected to a computer. To establish communication between the xScan and the computer, a programme was developed in C++. This programme is automatically executed through a user-friendly interface (UI) developed in MATLAB (MathWorks, Inc., Natick, MA, USA). The UI was programmed so that the ESUS is automatically configured within safe acoustic outputs.

**Table 2:** Manufacturing information of the main ESUS components

Component	Manufacturer	Model
Ophthalmic probe	IMASONIC SAS, Bourgogne-Franché-Comté, France	Custom made^a^
xScan	Tribosonics Ltd., Sheffield, England	Custom made^a^
Computer	ASUSTek Computer Inc., Nieuwegein, Netherlands	VM65N
UI	University of Coimbra	Custom made

^a^Equipment commercialized with a range of customer-defined features

Based on Annex VIII of Regulation (EU) 2017/745 of the European Parliament, the ESUS is classified as a type 2a medical device. This system has been developed in agreement with the Portuguese regulation, Law n° 145/2009 and with Regulation (EU) 2017/745 of the European Parliament.

#### ESUS procedures

i. Participant preparation: To reduce the discomfort generated by coupling the probe, topical anaesthesia (oxybuprocaine hydrochloride) will be applied before starting the acquisitions. However, if the participant has any known contraindication to this anaesthetic, a disposable neutral contact lens will be used as a replacement for the coupling medium between the probe and the cornea.
ii. The acquisitions are initiated through a starting button in the user interface, and the probe (previously clean and disinfected) is coupled to the eye after applying eye drops. The probe must be kept in place for a few seconds for automatic signal recording. During this period, signals are visualized in the user interface. Notably, as part of the clinical practice, the participant pupils will be previously dilated.
iii. Finally the probe is uncoupled, and the signals are saved in a directory along with other participant information.

#### Demographic and medical record

This information will be obtained from the patient record at the clinical centre with the purpose of confirming the inclusion/exclusion criteria and identifying biases. The data to be recorded are birth date, sex, occupation history, smoking and drinking habits and history of any ophthalmic or systemic diseases and associated treatments.

#### Slit lamp and LOCS III

Slit lamp is a noninvasive method in which cataracts are photographed by retroillumination. The cataract images will be classified according to the LOCS III for cataract type and severity [24]. Cataract classification by this method will be the comparator for this study, used to train the automatic classification system and evaluate its performance in humans.

#### Contrast sensitivity test

This test consists of observing images and identifying shapes of variable contrast. Contrast sensitivity is particularly affected in cataracts. Cases of incipient cataract, where slit lamp observation may not be sufficiently sensitive, may be identified by this method.

#### Specular microscopy

Specular microscopy is a noninvasive technique in which images are reconstructed from the specular reflection of an incident light beam magnified by a microscope. The density and shape of corneal endothelial cells will be evaluated by this method to diagnose corneal endothelial cell loss, which relates to excessive levels of phacoemulsification energy.

#### Optical coherence tomography (OCT)

OCT is a noninvasive diagnostic method, in which the reflection of a low-coherence light beam is used for image reconstruction. OCT will be used for measuring macular thickness to detect the presence of macular oedema, one of the possible postoperative complications of phacoemulsification surgeries [7]. This study is conducted as part of the clinical practice in patients submitted to phacoemulsification surgery.

#### Phacoemulsification surgery

The phacoemulsification procedure will be performed according to normal clinical practice. The maximum energy level used in the phacoemulsification surgery, the phacoemulsification time and possible surgical complications will be recorded to evaluate the appropriateness of phacoemulsification energy levels. This study will not interfere with the clinical surgery protocols.

#### Safety monitoring

The safety of the experimental medical device will be further monitored by clinical examination of the participants immediately after the ESUS study and by remote follow-up 1 week later. After this period, no adverse events related to the ESUS study will be expected.

Overall safety conditions will be monitored through the occurrence of unanticipated adverse events or serious adverse events. If these situations occur, the reportable adverse events will be communicated to the competent authorities according to current regulation, and the corrective actions will be implemented.

### Outcomes

#### Primary outcome

The presence of cataract is obtained with the ESUS.

Secondary outcomes are as follows:

i. Cataract type and severity estimated with ESUS based on the LOCS III
ii. Cataract hardness estimated with ESUS, measured in GPa
iii. OPE level estimated with ESUS based on the parameters indicated by the phacoemulsifier
iv. Number and severity of ESUS-related adverse events

#### Timelines and methods for outcomes recording

Study status: not yet recruiting

Starting date: October 2022

The total duration of the clinical study will be 15 months, and it will be divided into two phases:

- First phase: 9 months (6 months for recruitment and 3 months for participant follow-up).
- Second phase: 6 months (3 months for recruitment and 3 months for participant follow-up).

In the first phase, the cataract type and severity measured with slit lamp/LOCS III and contrast sensitivity will be used to train the algorithms for cataract detection and classification in humans. The surgical records (maximum phacoemulsification energy, phacoemulsification time and surgical complications), number and morphology of endothelial cells (measured by specular microscopy), the presence of macular oedema (assessed by OCT) and postoperative complications will support algorithm development for the estimation of OPE.

In the second phase, the cataract type and severity classification and OPE estimation will be implemented with the ESUS using the algorithms developed/trained in the first phase. The remaining study assessments will be used for preliminary performance evaluations. The methods for algorithm development are described in the “Statistical analysis” section.

### Statistical analysis

#### Feasibility assessment

The feasibility of the ESUS technique will be evaluated based on cataract detectability, cataract classification and OPE estimative performances and on the ESUS safety.

Cataract detectability will be considered suitable if at least 90% of the cases detected through slit lamp observations are identified by the ESUS.

The cataracts classification will be considered suitable if sensitivity levels higher than 90 %, with a maximum significance level of 5%, are reached.

The system efficacy for the OPE will be considered suitable if the prediction accuracy is higher than 90%.

Since the clinical procedure involved in data acquisition with the ESUS is similar to the used in biometry for IOL power calculation, the procedure viability is not object of the study.

The ESUS safety will be considered suitable under two conditions: (i) there are no occurrence of serious adverse event associated with ESUS whose causes cannot be rectified; (ii) the occurrence rate of mild to moderate ESUS-related adverse events is lower than 5%.

The feasibility study will be considered successful if at least the primary objective, detection of the presence of cataracts, and the secondary objective of cataracts classification (type and severity) are reached and if the safety of the ESUS technique is verified.

#### Cataract type and severity estimation

The automatic classification system will classify the cataract type (nuclear, cortical, or posterior subcapsular) and evaluate the severity.

From the acquired ultrasonic signals, several acoustic parameters in the time and frequency domains will be extracted. Then, more representative parameters will be selected through a principal component analysis and used for automatic classifier training (e.g. support vector machine, Bayes network and random forest). The performance of the different classifiers will be compared. The classification obtained by a trained specialist using the LOCS III will be used as a reference.

In the second study phase, a preliminary evaluation of the automatic classification performance will be conducted using a new dataset. It will be considered suitable if it reaches sensitivity levels higher than 90%, with a maximum significance level of 5%.

All the participants/eyes performing the ESUS study will be included in the analysis.

#### OPE estimation

To develop an OPE estimator, the acoustic parameters extracted from the ultrasonic signals in the first phase will be correlated with the maximum energy used in the phacoemulsification surgery for each participant eye. Only surgeries without surgical or postoperative complications will be considered. A confidence interval of 95% followed by multiple comparison correction will be used to assess the significance of the correlations.

In the second phase, the acoustic parameters extracted from the ultrasonic signals will be used to predict the safety and risk intervals of phacoemulsification energy based on the correlation curves. To evaluate the prediction accuracy, surgeons should initiate surgeries at minimum energy levels and increase them gradually until they reach an efficient energy level. Then, the maximum energy level applied during surgery and the occurrence of surgical and/or postoperative complications will be compared with the safe and risk energy intervals predicted with the correlation curves. The system efficacy will be considered suitable if the prediction accuracy is higher than 90%.

#### Interim analysis

An interim analysis will be performed between the end of the first phase of the study and the beginning of the second phase of the study.

The sponsor will continuously analyse safety data and will evaluate the results from the interim analysis. Based on the analyses, a consensus between the responsible investigator and the sponsor may end or suspend the study. The Portuguese National Authority for Medicaments and Health Products (INFARMED, I.P.) or the Portuguese National Ethical Committee for Clinical Research (CEIC) may also prematurely end the study.

## Ethics, safety and dissemination

### Ethical considerations

The protocol is based on respect for the life, health, well-being and privacy of the participants. The study will be conducted in accordance with the ethical principles stated in the Helsinki Declaration. The study protocol was approved by the INFARMED, I.P., the CEIC and the independent Ethical Committee of the Coimbra Surgical Centre.

### Patient and public involvement

The patients or the public were not involved in the design of the clinical study.

### Safety considerations

A safety evaluation of the ESUS has been previously reported [31]. The ESUS has been implemented in agreement with Portuguese Law n° 145/2009 and with Regulation (EU) 2017/745 of the European Parliament. Safety assessment was based on the standards DIN EN ISO 14971:2009-10 [32], IEC 60601-2-37 [33], IEC 62127-1 [34] and IEC 62359 [35]. Several potential risks were identified and managed, including the following:

i. Thermal and mechanical effects of ultrasound
ii. Excessive surface temperatures
iii. Electrical leakage
iv. Allergies
v. Mechanical risks
vi. Contamination
vii. Biocompatibility
viii. Configuration errors
ix. Electromagnetic interferences

Based on the ESUS risk assessment and management, there are no predictable adverse effects related to the use of the experimental medical device. Nonetheless, the sponsor will continuously monitor safety data and take the necessary measures to ensure participant safety. The remaining procedures to be implemented in the context of the study are noninvasive and will be carried out with certificated and commercial devices and by experienced specialists, not representing potential risks.

### Financing and insurance

The participants will not receive any monetary compensation or incur any expenses related to their participation in the study. However, they will be covered by an insurance policy from the HDI Global SE company.

### Protocol amendments

Any significant amendment in the protocol should be previously authorized by the sponsor and the competent authorities. If any protocol adjustment requires rapid execution due to safety reasons, it will be implemented without previous authorization and communicated a posteriori within no more than 10 business days.

### Dissemination

The results obtained in this study will be published in peer-reviewed journals and presented at national and international congresses. Study publications will be written in agreement between the sponsor and the principal investigator. Additionally, a patent for the ESUS is expected at the end of the study.

### Data management and confidentiality

All participants’ data will be recorded on individuals’ CRF in a legible and reliable way. The sponsor/representative should verify the complete and correct data.

Each participant will be identified by a unique numerical code of three digits. The principal investigator will be responsible for protecting the forms that identify the individual and relate them to the numerical code (i.e. the consent form and contact information pages of the CRF). The codified parts of the CRF will be transmitted to the sponsor.

A digital and a printed copy of the CRF will be kept to avoid accidental loss. All data collected during the study should be maintained at the investigational centre for a period of 25 years according to Regulation (EU) 536/2014 of the European Parliament.

The identity of the participants will not be revealed in any publication, in agreement with Regulation (EU) 2016/679 of the European Parliament.

### Roles and responsibilities

- Sponsor: University of Coimbra; contact name: Jaime Batista Santos, PhD, Department of Electrical and Computer Engineering, Faculty of Science and Technology, Pole II of the University of Coimbra, 3030-290, Coimbra, Portugal; phone: +351 239 796 263; and e-mail: jaime@deec.uc.pt
- Principal investigator: António Casa Nova Tavares Travassos, MD, Coimbra Surgical Centre, Dr. Manuel Campos Pinheiro street, n° 51, São Martinho do Bispo, 3054-089, Coimbra, Portugal
- Responsible investigator: Luís Miguel da Luz Caixinha Duarte, OD, PhD, University of Beira Interior, Department of Physics, 6291-001 Covilhã, Portugal

The clinical protocol (version n° 3, December 2019) was designed jointly by the sponsor and the principal investigator. The principal investigator and the clinical staff will conduct the participants’ recruitment and interventions in agreement with the protocol. The sponsor is responsible for the experimental device and data analysis. The sponsor will carry out monitoring activities, respond to the competent authorities, elaborate on reports and provide any other required documentation. The responsible investigator will coordinate and verify the correct performance of the clinical study; therefore, there will be no external data monitoring committee.

## Discussion

Preclinical studies showed that the experimental device presented positive performance for cataract classification and did not identify potential risks when used within the safety range by trained professionals. The present protocol refers to the first human pilot study of the experimental medical device ESUS. This study aims to evaluate the feasibility and safety of ESUS for quantitative and automatic cataract characterization and OPE estimation in human lenses. It is expected that the obtained results will be used in a larger clinical study to increase the dataset, to improve the performance of the algorithms for automatic classification and to train the algorithms for other causes of cataracts (in addition to age-related cataracts).

Several measures have been taken to minimize bias in the clinical study. The acquisition software was optimized to identify the correct probe positioning, thereby minimizing errors due to misalignment. The cataract group will be limited to age-related cataracts to reduce divergences in clinical conditions that could influence the results. A number of demographic and clinical information from participants will also be analysed for the identification of any additional source of bias.

The clinical study protocol presented here is a key piece of long-term translational research. The quantitative characterization of cataracts and estimation of OPE could significantly reduce postoperative complications and surgical times related to phacoemulsification surgeries, thus providing important social and economic benefits.

## Data Availability

Not applicable.

## Abbreviations

CEIC: National Ethical Committee for the Clinical Research
CRF: Case report form
ESUS: Eye Scan Ultrasound System
INFARMED, I.P.: National Authority for Medicaments and Health Products
IOL: Intraocular lens
LOCS III: Lens Opacity Classification System III
OCT: Optical coherence tomography
OPE: Optimal phacoemulsification energy
UI: User’s interface
